# P14 Improving adherence to and effectiveness of an adult critical care vancomycin continuous infusion protocol: a pilot quality improvement and administration data accuracy project

**DOI:** 10.1093/jacamr/dlac133.018

**Published:** 2023-01-19

**Authors:** Robert Oakley, Ha Trinh, Ting Yau, Sarah Korshid, Dagan Lonsdale

**Affiliations:** St George’s University Hospitals NHS Foundation Trust, St George’sUniversity of London; St George’s University Hospitals NHS Foundation Trust, St George’sUniversity of London; St George’s University Hospitals NHS Foundation Trust, St George’sUniversity of London; St George’s University Hospitals NHS Foundation Trust, St George’sUniversity of London; St George’s University Hospitals NHS Foundation Trust, St George’sUniversity of London

## Abstract

**Background:**

Vancomycin treats serious Gram-positive infections. In St George's Hospital (SGH) intensive care unit (ICU) settings, vancomycin is administered intravenously by continuous infusion. Steady-state serum concentrations are monitored daily with a 20–25 mg/L therapeutic target. Non-therapeutic concentrations are associated with patient harm and prolonged stay.^1^ A service evaluation revealed variable adherence to/effectiveness of the vancomycin prescribing/administration/monitoring protocol. Electronic prescribing and medicine administration (ePMA) system interface issues may have contributed.^2^ Consequently, multifaceted interventions were devised and piloted on General ICU.

**Objectives:**

To (i) improve combined protocol prescribing/administration/monitoring adherence; (ii) enhance therapeutic protocol dosing; and (iii) ascertain accuracy of patients’ paper drug administration (PDA) charts compared with the ePMA system.

**Methods:**

The quality improvement project (QIP) was approved by SGH clinical governance/audit teams. Over a 9 month period (September 2021 to May 2022) system/person-focused interventions were implemented. Protocol dosing^2^ was revised; introducing a >90 kg patient 2 g loading dose, new renal-function categories and an increased maintenance dose for creatinine clearance (CL_CR_) >90 mL/min. Protocol accessibility was increased via ePMA and CliniBee/Microguide app integration. Educational protocol presentations were incorporated into medical/nursing induction training. Vancomycin prescribing/administration/monitoring data for non-renal replacement patients during the intervention period, was extracted retrospectively from the ePMA system. This was compared with baseline informing ICU data collected July 2020 to July 2021.^2^ Patient's drug administration accuracy data (PDA charts/ePMA system) was extracted retrospectively from January to May 2022 and analysed.

**Results:**

Compared with baseline, the proportion of patients receiving per protocol prescribing/administration of vancomycin loading/maintenance doses with daily monitoring increased [39% (7/18) to 68% (15/22)]. Within 48 h 54% (7/13) of vancomycin serum concentrations in all patients were therapeutic, demonstrating a baseline increase of 21% (3/9 to 7/13). In per protocol treated patients, serum concentrations increased 16% (2/7 to 4/9) therapeutically, decreased 20% (3/7 to 2/9) supra-therapeutically and increased 4% (2/7 to 3/9) sub-therapeutically. Supra-therapeutic concentrations were associated with CL_CR_ <50 mL/min. Sub-therapeutic concentrations were associated with CL_CR_ >90 mL/min and obesity. Compared with the ePMA system, there was 38% (5/13) less PDA charts recording both loading/maintenance doses. Administration time differences >60 min were recorded for 38% (3/8) of loading and 31% (4/13) maintenance doses.

**Discussion:**

Staff turnover periods were associated with decreased protocol compliance. Further education is required around prescribing/administration of standardized vancomycin infusion bags, with associated rate changes. Integration of pharmacists into daily Microbiology ward rounds may alleviate these issues. Higher 20 mg/kg loading doses for >100 kg patients and maintenance dose revisions may reduce non-therapeutic concentrations.^3^ Multidirectional variation in vancomycin administration timings recorded between PDA charts/ePMA system, requires further investigation. Infusion-pump data may offer the most accurate administration time for calculating pharmacokinetic variables.

**Conclusions:**

Multifaceted interventions were successful at improving adherence to/effectiveness of the vancomycin protocol. Findings will inform QIP roll-out across all three SGH ICUs, which will incorporate infusion-pump data collection to facilitate pharmacokinetic modelling. This will inform local dosing strategies and research into patient variability.
