# Correction to: Improving adherence to and effectiveness of an adult critical care vancomycin continuous infusion protocol: a pilot quality improvement and administration data accuracy project

**DOI:** 10.1093/jacamr/dlad075

**Published:** 2023-06-08

This is a correction to: Rob Oakley and others, P14 Improving adherence to and effectiveness of an adult critical care vancomycin continuous infusion protocol: a pilot quality improvement and administration data accuracy project, *JAC-Antimicrobial Resistance*, Volume 5, Issue Supplement_1, February 2023, dlac133.018, https://doi.org/10.1093/jacamr/dlac133.018

In the originally published version of this manuscript, the author and affiliations list details were incomplete.

These should read:

“Robert Oakley^1^, Ha Trinh^1^, Ting Yau^1^, Sarah Korshid^1^, Dagan Lonsdale^1^

1 St George's University Hospitals NHS Foundation Trust, St George's University of London” instead of:

“Rob Oakley^1^

1 St George’s University Hospitals NHS Foundation Trust, UK”.

These details have been emended in the paper online.

